# Prognostic value of post-discharge depression in patients recently hospitalized with acute heart failure

**DOI:** 10.3389/fcvm.2022.858751

**Published:** 2022-08-02

**Authors:** Junlei Li, Chao Jiang, Rong Liu, Yiwei Lai, Li Li, Xiaoyan Zhao, Xiaofang Wang, Ling Li, Xin Du, Changsheng Ma, Jianzeng Dong

**Affiliations:** ^1^Department of Cardiology, The First Affiliated Hospital of Zhengzhou University, Zhengzhou, China; ^2^Department of Cardiology, Beijing AnZhen Hospital, National Clinical Research Centre for Cardiovascular Diseases, Beijing Advanced Innovation Center for Big Data-Based Precision Medicine for Cardiovascular Diseases, Capital Medical University, Beijing, China; ^3^Heart Health Research Center (HHRC), Beijing, China; ^4^The George Institute for Global Health, The University of New South Wales, Sydney, NSW, Australia

**Keywords:** acute heart failure, depression, heart failure hospitalization, early post-discharge period, heart failure with preserved ejection fraction

## Abstract

**Background:**

Depression is a prevalent comorbidity in patients with heart failure (HF). However, data regarding the prognostic significance of depression during the early post-discharge period in patients hospitalized with acute HF, regardless of left ventricular ejection fraction (LVEF), were scarce.

**Methods and results:**

The Heart Failure Registry of Patient Outcomes (HERO) study is a prospective, multicenter study of patients hospitalized with acute HF in China. At the first follow-up after discharge (median 4.0, interquartile range [IQR]: 2.4–6.1 weeks), depressive symptoms over the past 2 weeks were assessed using the Patient Health Questionnaire-9 (PHQ-9). Of 3,889 patients, 480 (12.3%) patients had depression (PHQ-9 score ≥ 10). A total of 3,456 patients (11.4% with depression) were included in the prospective analysis. After a median follow-up of 47.1 weeks (IQR: 43.9, 49.3) from the first follow-up, 508 (14.7%) patients died, and 1,479 (42.8%) patients experienced a composite event (death or HF rehospitalization). Cox proportional hazards models were used to assess the association of post-discharge depression with adverse events. After adjustment, post-discharge depression was associated with an increased risk of all-cause mortality (hazard ratio [HR] 2.38 [95% confidence interval (CI): 1.93–2.94]; *p* < 0.001) and the composite event (HR 1.78 [95% CI: 1.55–2.05]; *p* < 0.001). A per scale point increase in PHQ-9 score (ranging from 0 to 27 points) was associated with a 7.6% increase in all-cause mortality (HR 1.08 [95% CI: 1.06–1.09]; *p* < 0.001). In the subgroup analysis, the association between depression and the composite event was significantly stronger in relatively younger patients (< 75 vs. ≥ 75 years; p for interaction = 0.011), and the association between depression and all-cause mortality was significantly stronger in patients with preserved ejection fraction than in those with reduced ejection fraction (*p* for interaction = 0.036).

**Conclusion:**

Post-discharge depression in patients recently hospitalized with acute HF is associated with an increased risk of adverse events, regardless of LVEF. Screening for depressive symptoms during the early post-discharge period may help to better identify high-risk patients and tailor patient management. Further studies are needed to determine how regular depression screening can help improve patient management and clinical outcomes.

## Introduction

Depression is a prevalent comorbidity in patients with heart failure (HF) and is associated with impaired quality of life and increased risk of hospitalization and mortality (1–3). Most previous studies on depression in HF have focused on patients with chronic HF with reduced ejection fraction (HFrEF) from Western and other developed countries (3, 4). Following hospitalization for acute HF, patients are at high risk of readmission and mortality (5, 6). Approximately 30% of patients are re-hospitalized within 2–3 months of discharge, and mortality can reach 15% during this period (7). High rates of events are mainly driven by a subgroup of high-risk patients (8, 9). It is necessary to stratify patients to identify those at high risk of adverse events and potentially improve outcomes. Screening for depressive symptoms during the early post-discharge period can be easily achieved using self-reported questionnaires over the phone in minutes (10). However, the prognostic significance of depression during the early post-discharge period in patients recently hospitalized with acute HF is unknown. In addition, nearly half of patients with HF have a normal left ventricular ejection fraction (LVEF) (11). Nevertheless, data on the associations between depression and HF with preserved ejection fraction (HFpEF) are limited (4). There are substantial differences in the clinical spectrum and pathophysiological mechanisms between HFpEF and HFrEF (11). However, it is unclear whether there is an interaction between LVEF and depression on clinical outcomes.

The prevalence of depression in the general population varies significantly between countries and regions. In the World Mental Health Survey conducted by the World Health Organization, the lifetime prevalence of any mood disorder in the United States was approximately six times higher than in China (21.4 vs. 3.6%, respectively) (12). Similarly, two recent large-scale cross-sectional studies (13, 14) also showed a 5-fold difference in the lifetime prevalence of major depressive disorder between the United States and China (20.6 vs. 3.9%, respectively). A cross-sectional study (15) performed in a Chinese community-dwelling population reported that 12.0% of women and 7.7% of men with chronic HF had depression. To our knowledge, no large-scale prospective studies have been conducted on depression in patients with HF in China. Therefore, we investigated the prevalence, predictors, and prognostic value of post-discharge depression in a prospective cohort of patients hospitalized with acute HF in China.

## Methods

### Ethical considerations

All procedures performed in studies involving human participants were in accordance with the ethical standards of the institutional and/or national research committee and with the 1964 Declaration of Helsinki and its later amendments or comparable ethical standards. The study was approved centrally by the Ethics Committee on Scientific Research and Clinical Trials at The First Affiliated Hospital of Zhengzhou University (in September 2017; approval number: 2014SY-079), and by the local Health Research Ethics Board of each participating hospital. All patients provided written informed consent.

### Study population

The Heart Failure Registry of Patient Outcomes (HERO) study is a prospective, longitudinal, multicenter study aimed at determining the profile, management, and 1-year outcomes of patients hospitalized with acute HF in China (16). Adult (≥18 years) patients admitted to 73 participating hospitals with a primary admission diagnosis of acute HF during the defined period were consecutively recruited. The diagnosis of acute HF was confirmed by trained cardiologists, and patients with an incorrect admission diagnosis were excluded. From 10 November 2017 to 4 November 2018, 5,620 patients hospitalized with acute HF were enrolled. Data on socio-demographic characteristics, medical history, lifestyle, self-reported health status, clinical characteristics at admission, laboratory tests, treatments, and hospital course were collected. Only patients discharged alive who consented to follow-up calls were included. Standardized follow-up calls were made by trained nurses. Follow-up calls were scheduled at 2 weeks and 3, 6, and 12 months after discharge, or until death or withdrawal.

### Depressive symptoms evaluation

At the first follow-up after discharge, the Patient Health Questionnaire-9 (PHQ-9) was administered by trained nurses over the phone to assess depressive symptoms. PHQ-9 is a self-reported questionnaire, containing 9 items based on the Diagnostic and Statistical Manual, to determine the presence and severity of depressive symptoms over the past 2 weeks (10). Each depressive symptom score ranges from 0 (not at all) to 3 (nearly every day), with total scores ranging from 0 to 27. PHQ-9 scores of 0–4, 5–9, 10–19, and 20–27 represent minimal, mild, moderate, and severe depression, respectively. A PHQ-9 score ≥ 10 has a sensitivity and specificity of 88% for diagnosing major depressive disorder (10). PHQ-9 is an effective tool for assessing depressive symptoms in patients with HF (17) and in the elderly from China (18). In this study, a PHQ-9 score ≥10 was defined as clinical depression.

### Clinical outcomes

The primary outcomes were all-cause mortality and the composite of death or HF rehospitalization (determined from the time of post-discharge depression screening). Vital status and, if applicable, the cause of death of patients who provided consent but were lost to follow-up were determined using administrative data (16).

### Statistical analysis

Categorical variables were presented as frequencies and percentages and compared using the chi-square test. Continuous variables were presented as mean ± standard deviation, or median and interquartile range, and compared using the *t*-test or Mann–Whitney *U* test, as appropriate. A two-sided *p* < 0.05 was considered statistically significant. The baseline characteristics of patients with and without depression were compared. Multiple logistic regression analysis was performed using adjusted variables with *p* < 0.1 in the comparison of baseline characteristics. The outcome of the logistic regression analysis was depression at the first follow-up call. Cox proportional hazards regression analysis was performed to evaluate the associations between depression and adverse events. To evaluate the dose-dependent relationship between depressive symptoms and adverse events, we also included PHQ-9 scores as a continuous variable in the Cox regression analysis. Potential confounding variables were adjusted based on univariate analysis (*p* < 0.1) and clinical knowledge. The adjusted variables in the multivariate analysis included age; sex; body mass index (BMI); systolic blood pressure; current smoking status; coronary artery disease; diabetes mellitus; chronic obstructive pulmonary disease (COPD); anemia; estimated glomerular filtration rate; serum sodium concentration; in-hospital LVEF (< 40%, 40–49%, ≥ 50%); renin-angiotensin system (RAS) inhibitor, beta-blocker, mineralocorticoid receptor antagonist, and statin use at discharge; and hospital levels. Anemia was defined as hemoglobin < 13 g/dl in men or hemoglobin < 12 g/dl in women (19). Multiple imputations (Markov chain Monte Carlo method) were used to account for missing data. A total of 40 imputed datasets were generated. In-hospital N-terminal pro-B-type natriuretic peptide (NT-proBNP) levels were measured in 1,890 (54.7%) of 3,456 patients. Brain natriuretic peptide levels were available for 880 (25.5%) patients without NT-proBNP measurements. In another sensitivity analysis, brain natriuretic peptide and NT-proBNP levels were log-transformed and standardized, respectively. They were then combined into a new variable, NPs, as described previously (Supplementary Material) (20). In the NPs model, NPs levels (presented as *z* scores, another indicator of HF severity) were included in the adjusted model to further test the independence of the association between depression and adverse events. Subgroup analysis was performed to explore the interaction between depression and adverse events stratified by age (< 75 vs. ≥75 years), sex, in-hospital LVEF (<40%, 40–49%, and ≥50%), and New York Heart Association (NYHA) class (IV vs. III). Statistical analysis was performed using IBM SPSS Statistics (version 26).

## Results

### Prevalence and characteristics of patients with depression

**FIGURE 1:**
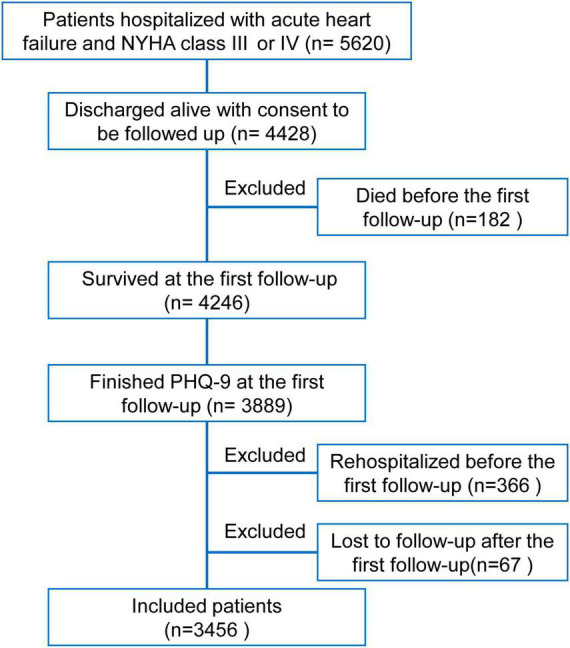
Flowchart of this study. Of 5,620 patients hospitalized with acute heart failure and New York Heart Association class III or IV in the Heart Failure Registry of Patient Outcomes study, 4,428 patients were discharged alive with consent to be followed up. After excluding 182 patients who died before the first follow-up, 3,889 of 4,246 survived patients had valid patient health questionnaire-9 scores. To avoid the effects of repeated hospitalizations in a short period on assessing depressive symptoms, 366 patients who had already been rehospitalized before the first follow-up were excluded. Meanwhile, 67 patients lost to further follow-up calls after the first follow-up were excluded. Finally, a total of 3,456 patients were included in the present analysis.

Of 5,620 patients hospitalized with acute HF (NYHA class III or IV) in the HERO study, 4,428 patients were discharged alive with consent to be followed up. One hundred and eighty-two patients who died before the first follow-up were excluded. Of 4,246 survivors, 3,889 had valid PHQ-9 sores. In the prospective analysis, 366 patients who were re-hospitalized before the first follow-up and 67 patients who were lost to follow-up after the first follow-up call were excluded. Finally, a total of 3,456 patients were included (Figure 1).

**FIGURE 2:**
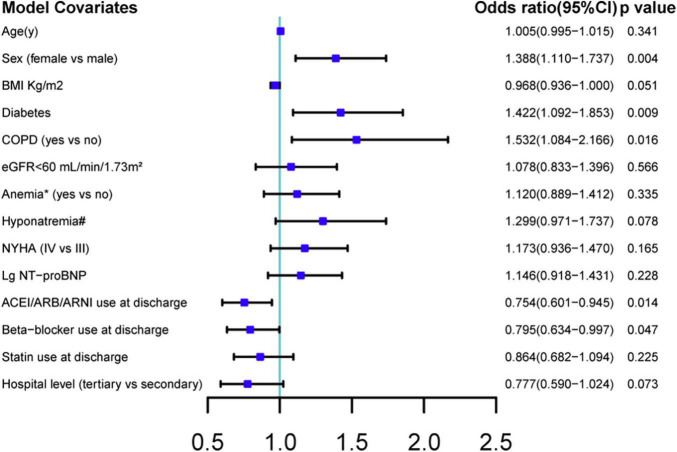
Multiple logistic regression analysis of predictive factors associated with post-discharge depression. BMI, body mass index; NYHA, New York Heart Association; COPD, chronic obstructive pulmonary disease; eGFR, estimated glomerular filtration rate; ACEI, angiotensin-converting enzyme inhibitors; ARB, angiotensin receptor blockers; ARNI, angiotensin receptor-neprilysin inhibitors; Lg NT-proBNP, log-transformed values of N-terminal pro-B-type natriuretic peptide. CI: confidence interval. Factors in baseline characteristics with a p-value < 0.1 were included. Anemia, hemoglobin < 13 g/dl in male patient or hemoglobin < 12 g/dl in female patient. Hyponatremia, serum sodium concentration < 135 mmol/L.

The median first follow-up was 4.0 (interquartile range: 2.4–6.1) weeks after discharge. Of 3,889 patients with available PHQ-9 scores, 480 (12.3%) patients had depression (PHQ-9 score ≥ 10). In the prospective cohort, 393 (11.4%) of 3,456 patients had depression. As shown in Table 1, patients with depression had the following characteristics: female sex, older age, lower BMI, decreased estimated glomerular filtration rate (≤60 ml/min/1.73 m^2^), higher NYHA class (IV vs. III), higher NT-proBNP levels, lower serum sodium concentration, diabetes mellitus, COPD, and anemia. RAS inhibitor, beta-blocker, and statin use at discharge were lower in patients with depression. Patients with depression were also more likely to be hospitalized in a secondary hospital than in a tertiary hospital. In the adjusted model, female sex, COPD, and diabetes mellitus were independent risk factors for depression; The use of RAS inhibitor and beta-blocker at discharge was independently associated with less depression (Figure 2).

**TABLE 1:** Baseline characteristics.

Variables	No depression [PHQ-9 score<10]	Depression [PHQ-9 score ≥ 10]	*P*-value
Overall *n*, (%)	3063 (88.6)	393 (11.4)	-
**Socio-demographic**			
Age, year, mean (SD)	71.54 (12.25)	73.87 (11.26)	< 0.001
Female sex, *n* (%)	1502 (49.0)	230 (58.7)	< 0.001
Education, Elementary school or below, *n* (%)	2048 (76.4)	277 (78.5)	0.422
income < 30k RMB per year, *n* (%)	1751 (67.3)	237(69.3)	0.457
no/low-coverage insurance#, *n* (%)	2292 (75.9)	301 (77.8)	0.448
Tertiary-level hospital (versus secondary), *n* (%)	743 (24.3)	72 (18.3)	0.010
Current smoking, *n* (%)	256 (8.4)	28 (7.2)	0.493
Current drinking, *n* (%)	142 (4.7)	12 (3.1)	0.192
**Clinical features**			
BMI, kg/m^2^, mean (SD)	23.43 (4.39)	22.64 (3.83)	0.001
SBP, mmHg, mean (SD)	135.51 (24.82)	134.81 (24.73)	0.596
Heart rate, mean (SD)	87.67 (22.52)	88.32 (22.23)	0.590
In-hospital LVEF, median (IQR)	52 (39, 60)	53 (42, 60)	0.499
In-hospital LVEF < 50%, *n* (%)	795 (44.0)	94 (43.5)	0.942
NYHA class IV (versus III), *n* (%)	1385 (45.2)	209 (53.2)	0.003
BNP, pg/mL, median (IQR)	936 (317-3239)	1102 (371-3575)	0.690
NT-proBNP, pg/mL, median (IQR)	2735 (854-6840)	3421 (902-9077)	0.029
eGFR < 60mL/min/1.73m^2^, *n* (%)	816 (28.8)	128 (35.7)	0.008
Anemia*, *n* (%)	1369 (46.3)	197 (53.7)	0.008
LDL-C, mmol/L, median (IQR)	2.14 (1.67, 2.78)	2.20 (1.71, 2.74)	0.719
Serum sodium, mmol/L, mean (SD)	139.27 (4.59)	138.22 (4.92)	< 0.001
Serum potassium, mmol/L, mean (SD)	4.15 (0.65)	4.16 (0.67)	0.827
**Medical history**			
Hypertension, *n* (%)	1489 (48.8)	185 (47.2)	0.555
Diabetes, *n* (%)	585 (19.2)	93 (23.7)	0.036
CAD, *n* (%)	873 (28.7)	120 (30.7)	0.441
MI	411 (13.4)	63 (16.0)	0.162
PCI	213 (7.0)	27 (6.9)	0.943
CABG	53 (1.7)	7 (1.8)	0.839
Valvular heart disease, *n* (%)	753 (24.8)	86 (22.1)	0.260
Congenital heart disease	l33 (1.1)	6 (1.5)	0.520
COPD, *n* (%)	240 (7.9)	47 (12.1)	0.006
Atrial fibrillation, *n* (%)	801 (26.2)	110 (28.2)	0.428
Cerebrovascular disease, *n* (%)	445 (15.3)	54 (14.4)	0.702
**Treatment at discharge**			
Renin-angiotensin system inhibitors, *n* (%)	1460 (48.2)	148 (38.0)	< 0.001
β-blockers, *n* (%)	1653 (54.4)	175 (45.0)	0.001
MRA, *n* (%)	2195 (72.2)	291 (74.8)	0.305
Diuretics, *n* (%)	1818 (60.1)	230 (59.7)	0.912
Digoxin, *n* (%)	654 (21.5)	90 (23.2)	0.472
Statin, *n* (%)	2137 (70.4)	250 (64.4)	0.016

SD, standard deviation; IQR, interquartile range; SBP, systolic blood pressure; BMI, body mass index; LVEF, left ventricular ejection fraction; CAD, coronary artery disease; MI, myocardial infarction; PCI, percutaneous coronary intervention; CABG, coronary artery bypass graft; NYHA, New York Heart Association; BNP, brain natriuretic peptide; NT-proBNP, N-terminal pro-B-type natriuretic peptide; eGFR, estimated glomerular filtration rate; COPD, chronic obstructive pulmonary disease; MRA, mineralocorticoid receptor antagonists. *Anemia, hemoglobin < 13 g/dl in male patient or hemoglobin < 12 g/dl in female patient. #New rural cooperative medical scheme was defined as low-coverage insurance. eGFR was calculated with the Chronic Kidney Disease Epidemiology Collaboration (CKD-EPI) equation.

### Clinical outcomes in patients with depression

**FIGURE 3:**
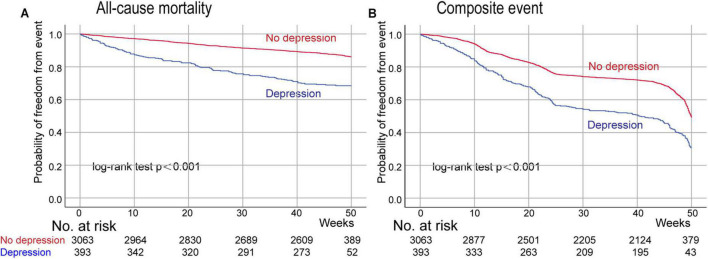
Kaplan–Meier curves for all-cause mortality and the composite event (death or HF rehospitalization) according to post-discharge depression. Day 0 is the time of the first follow-up after discharge. Panel **(A)**, the probability of freedom from all-cause mortality; Panel **(B)** the probability of freedom from the composite of death or HF rehospitalization.

**FIGURE 4:**
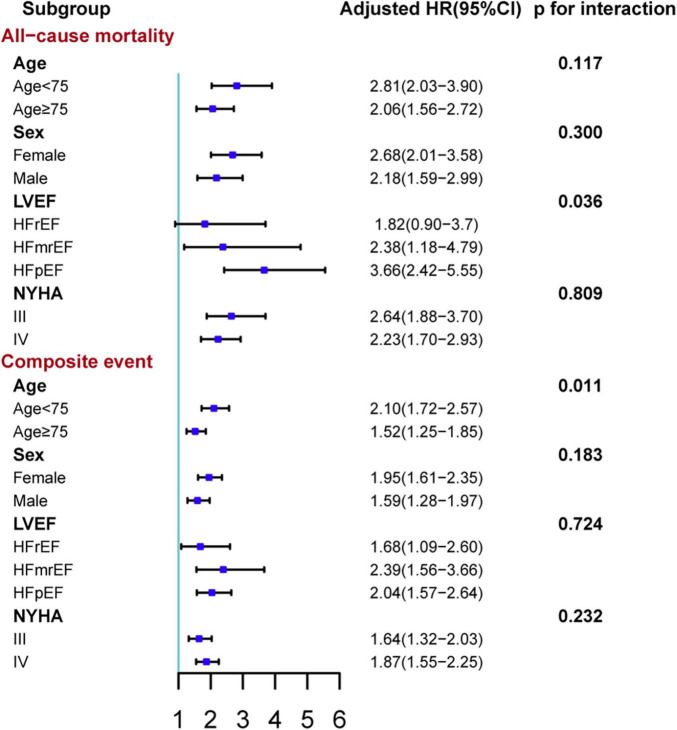
Subgroup analyses of the association between depression and adverse outcomes stratified by age, sex, in-hospital ejection fraction, and NYHA class. The composite event was time to death or HF rehospitalization. Adjusted variables in the Cox proportional hazard model: age, sex, body mass index, systolic blood pressure, left ventricular ejection fraction, current smoker, diabetes, chronic obstructive pulmonary disease, coronary heart disease, anemia, estimated glomerular filtration rate, serum sodium concentration, New York Heart Association class, and the use of renin-angiotensin system inhibitors, β-blockers, mineralocorticoid receptor antagonists and statin at discharge, and hospital levels. Anemia was defined as hemoglobin <13 g/dl in male patient or hemoglobin <12 g/dl in female patient. HFrEF, heart failure with decreased ejection fraction; HFmrEF, heart failure with mildly reduced ejection fraction; HFpEF, heart failure with preserved ejection fraction. HR, hazard ratio; CI: confidence interval.

After a median follow-up of 47.1 (interquartile range: 43.9–49.3) weeks from the first follow-up (Figure 3), 508 (14.7%) patients died and 1,479 (42.8%) patients experienced a composite event (death or HF rehospitalization). In the unadjusted, adjusted, and NPs models, post-discharge depression was associated with an increased risk of all-cause mortality and the composite of death or HF rehospitalization (all *p* < 0.001; Table 2). After adjustment, depression was associated with a 138% increase in all-cause mortality (hazard ratio [HR] 2.38 [95% confidence interval (CI): 1.93–2.94]) and a 78% increase in the composite event rate (HR 1.78 [95% CI: 1.55–2.05]). A per scale point increase in PHQ-9 score was associated with a 7.6% increase in all-cause mortality (HR 1.08 [95% CI: 1.061–1.091]; *p* < 0.001) and a 4.6% increase in the composite event rate (HR 1.05 [95% CI: 1.036–1.056]; *p* < 0.001) after adjustment (Supplementary Material). In the sensitivity analysis, the results of the complete case analysis and handling missing data with multiple imputations were consistent (Supplementary Material). In the subgroup analysis, the association between depression and the composite event was significantly stronger in relatively younger patients (<75 vs. ≥ 75 years; *p*- for interaction = 0.011), and the association between depression and all-cause mortality was significantly stronger in patients with HFpEF than in those with HFrEF (*p*- for interaction = 0.036; Figure 4).

**TABLE 2:** The associations of depression with all-cause mortality and the composite event (death or HF rehospitalization).

	All-cause mortality			Composite event		
		
	HR	95%CI	*P* value	HR	95%CI	*P* value
Unadjusted model	2.80	2.28-3.43	< 0.001	1.92	1.68-2.20	<0.001
Adjusted model*	2.38	1.93-2.94	< 0.001	1.78	1.55-2.05	<0.001
NPs Model#	2.35	1.90-2.90	< 0.001	1.77	1.54-2.04	<0.001

*Adjusted variables: age, sex, body mass index, systolic blood pressure, current smoker, diabetes, chronic obstructive pulmonary disease, coronary heart disease, anemia, estimated glomerular filtration rate, serum sodium concentration, in-hospital left ventricular ejection fraction group (<40%, 40–49%, ≥50%), New York Heart Association class, and the use of renin-angiotensin system inhibitors, β-blockers, mineralocorticoid receptor antagonists and statin at discharge, and hospital levels. Anemia was defined as hemoglobin <13 g/dl in male patient or hemoglobin <12 g/dl in female patient. #Adjusted variables in the NPs model: all aforementioned variables and additional adjustments for NPs. Brain natriuretic peptide and N-terminal pro-B-type natriuretic peptide values were log-transformed and standardized, respectively. Then, they were combined into a new variable, NPs levels. NPs levels (presented as z score), as another adjusted variable, was added to the adjusted model. HF, heart failure; HR, hazard ratio; CI: confidence interval.

## Discussion

In this large-scale prospective study of patients recently hospitalized with acute HF, 12.3% of patients had depression in the early post-discharge period. Most were undiagnosed and untreated. Female sex, older age, lower BMI, lower serum sodium concentration, worse HF severity, more comorbidities, and not using RAS inhibitors, beta-blockers, or statins at discharge were associated with depression. Post-discharge depression in patients recently hospitalized with acute HF was significantly and independently associated with an increased risk of all-cause mortality and the composite of death or HF rehospitalization. The association between post-discharge depression and adverse events was significantly stronger in relatively younger patients and in patients with HFpEF.

Previous studies in Western countries reported that the prevalence of depression in patients with HF was 20–30%, approximately three times the prevalence of major depressive disorder in the general population (2, 14). Depression was also more common in patients with a higher NYHA class, and inpatients were more likely to be depressed than outpatients (2). In this study, we assessed depressive symptoms during the early post-discharge period. This inevitably excluded patients who died before the first follow-up and those lost to the first follow-up. Discharged patients tended to have improved symptoms after in-hospital therapy. The timing of depression assessment and patient selection bias may partly explain the relatively lower prevalence in our study. Of 480 patients with depression, only seven had a history of mental illness, and only four had been treated with antidepressants, indicating an extreme lack of awareness and treatment of depression in patients with HF in China. The under-diagnosis and under-treatment of depression in patients with cardiovascular disease have been reported previously. One study showed that only 11% of patients with depression received appropriate treatment (21). The socioeconomic status (22) and cultural characteristics (e.g., more stigma toward mental illness (23)) of China may have exacerbated this problem. In the China Mental Health Survey, only 0.5% of individuals with major depressive disorder were treated adequately (24). Depression severely decreases the quality of life and increases morbidity and mortality (25). It was estimated that there were 13.7 million patients with HF in China (26), suggesting a large number of patients with undiagnosed depression that warrants attention.

The reasons why depression is prevalent in patients with HF and correlates with poor prognosis are complex and multifactorial (4). Several shared pathological mechanisms between HF and depression have been proposed, including higher levels of inflammatory markers, abnormal activation of the hypothalamic-pituitary-adrenal axis, autonomic dysfunction, and lifestyle factors (4, 27). Consistent with a previous study of chronic HF (4), our study showed that female sex, worse HF severity (higher NYHA class and NT-proBNP levels), and more comorbidities were significant predictors of post-discharge depression in univariate analysis. Depression is both a contributing factor and a consequence of the exacerbation of HF (4). The higher symptom burden, poorer quality of life, and increased activation of shared pathophysiological pathways between depression and HF may account for the higher rate of depression in patients with more severe HF (28). Previous studies reported that higher BMI and younger age were associated with depression in patients with chronic HF (29, 30) and in the general population (14, 31), whereas our findings were the opposite. These differences may partly be explained by the worse HF severity in patients with lower BMI and older age in this study, as they also had a much higher mortality rate during follow-up (Supplementary Material). Theoretically, patients with stable and chronic HF may be more similar to the general population (31). Conversely, in patients recently hospitalized with acute HF, secondary depression due to severe somatic disorders (32) may surpass the effects of a higher BMI and younger age. In this study, COPD and diabetes mellitus were independent predictors of post-discharge depression. These comorbidities may further increase the symptom burden and reduce the quality of life. Patients with COPD (33) and diabetes mellitus (34) tend to have more chronic inflammation, which may be an important mechanism for the increased risk of post-discharge depression. The use of RAS inhibitor and beta-blocker at discharge was independently associated with less post-discharge depression after adjustment. Previous studies have reported that abnormal RAS activation and increased sympathetic tone may contribute to the pathophysiological mechanism of depression in patients with HF (35, 36). Our findings support this hypothesis.

The early post-discharge period after hospitalization for acute HF carries a high risk of adverse events, and post-discharge follow-up during this period is recommended by the current European Society of Cardiology guidelines (7, 11). Our findings highlight the importance of screening for depressive symptoms in this vulnerable phase, and monitoring has significant and independent value for risk stratification. There is currently no clear consensus regarding the treatment of depression in patients with HF (11). Whether depression is a treatable target in HF or serves as a marker of HF severity remains to be determined. Potential interventions for patients with HF and post-discharge depression include treatment for HF and treatment for depression. Depressed patients tend to have lower adherence to HF treatment and poorer self-management and may require more intensive monitoring by clinicians (4). Because of the higher risk of adverse events in patients with HF and depression, a more aggressive treatment strategy for HF may provide a greater benefit. Selective serotonin reuptake inhibitors (SSRI) are widely used as antidepressants (37). However, SSRI failed to improve depression or the prognosis of HF in patients with HFrEF in two randomized controlled trials (38, 39). In the MOOD-HF trial, although escitalopram did not improve depressive symptoms more significantly than placebo, patients in both study arms showed a substantial improvement in depressive symptoms compared with baseline after nurse-coordinated disease management and optimization of HF drug therapy (38). This suggests that optimal treatment of HF may be the key to reducing depressive symptoms. Other studies have shown that exercise training (40), cognitive-behavioral therapy (41), telemedical interventional monitoring (42), and palliative care (43) improve depressive symptoms in patients with HF. These additional interventions may be beneficial for depressive symptoms in post-discharge patients with depression after the optimization of guideline-directed medical therapy for HF. However, the clinical significance of the reduction in depressive symptoms requires further research.

Previous studies on depression in patients with HFpEF are limited. This study is the first to report an interaction between post-discharge depression and in-hospital LVEF on mortality in patients recently hospitalized with acute HF. Our results were inconsistent with those of a recent *post hoc* analysis of the TOPCAT trial, in which no significant association was found between baseline depression and mortality in patients with chronic HFpEF after a mean follow-up of 3.3 years (44). In the TOPCAT trial, patients with a life expectancy of < 3 years were excluded. Two-thirds of patients had NYHA class I or II symptoms, indicating that the enrolled patients were more likely to have relatively stable HFpEF at baseline (45). Conversely, patients in the HERO study had NYHA class III or IV symptoms at enrolment. Worse HF severity, higher rates of adverse events, shorter time intervals between baseline evaluation of depressive symptoms and the occurrence of events, and more HF-related depression may explain the significant associations in the HERO study. In the TOPCAT trial, worsening depressive symptoms at 12 months were associated with an increased risk of all-cause mortality (HR 1.82 [95% CI: 1.13–2.93) (30), suggesting that depression may be a marker of the deterioration of chronic HFpEF. Our study extended the findings of the TOPCAT trial and indicated that, in patients who have recently experienced acute decompensated HF, depression is a stronger predictor of mortality in HFpEF than in HFrEF.

## Limitations

There are several limitations to this study. First, although PHQ-9 is an effective tool with high sensitivity and specificity, it remains a self-reported questionnaire, not diagnostic criteria. PHQ-9 was administered by trained nurses over the phone, not strictly self-administered. Some patients may have been afraid or unwilling to share their poor mental state when answering questions posed by nurses. Second, baseline characteristics were collected during hospitalization in the HERO study, while depressive symptoms were assessed at the first follow-up. The timing discrepancy may have introduced potential confounders and influenced our results. Because depressive symptoms were assessed at the first follow-up, only patients who (i) were discharged, (ii) did not die early after discharge, (iii) had not been rehospitalized before the first follow-up, and (iv) were prepared to answer the PHQ-9 were included. Therefore, selection bias may have influenced the results. Third, the severity of depressive symptoms in patients with HF may have fluctuated. Some studies showed that the improvement or worsening of depression over time was associated with HF outcomes (30, 40). In the HERO study, we only assessed baseline depressive symptoms; therefore, we could not evaluate the associations between dynamic changes in depressive symptoms and clinical outcomes. Finally, the enrolment of patients in the HERO registry was limited to Henan Province, China, with a population of 100 million. Further validation is required in more diverse populations.

## Future directions

Post-discharge depression is common in patients recently hospitalized with acute HF and is associated with adverse events. Unfortunately, depression in these patients often goes undiagnosed and untreated. Therefore, clinicians should pay more attention to the post-discharge mental health status of patients with HF. Because traditional antidepressants failed to improve depressive symptoms more significantly than placebo, further studies are needed to determine what can be done to relieve depressive symptoms and improve HF outcomes. Most previous studies on the treatment of depression in HF have focused on patients with HFrEF. Our study suggests that the association between depression and adverse events may be even stronger in patients with HFpEF than in those with HFrEF; therefore, future studies involving the treatment of depression in patients with HFpEF are necessary.

## Conclusion

Post-discharge depression in patients recently hospitalized with acute HF is associated with an increased risk of adverse events, regardless of LVEF. Screening for depressive symptoms during the early post-discharge period may help to better identify high-risk patients and tailor patient management. Further studies are needed to determine how regular depression screening can help improve patient management and clinical outcomes.

## Data availability statement

The original contributions presented in the study are included in the article/Supplementary material, further inquiries can be directed to the corresponding author/s.

## Ethics statement

The studies involving human participants were reviewed and approved by the Ethics Committee on Scientific Research and Clinical Trials at the First Affiliated Hospital of Zhengzhou University (in September 2017; approval no. 2014SY-079). Written informed consent to participate in this study was provided by the patients.

## Author contributions

JD, XD, and JL contributed to the conception and design of the study. XW, RL, XZ, LinL, and LiL participated in the collection of samples and data. RL and YL performed the statistical analysis and all the results were checked by CJ and LiL. JL wrote the first draft of the manuscript. LinL, YL, XZ, XW, and CJ wrote sections of the manuscript. CM, XD, and JD were critically revised the manuscript. All authors contributed to the article and approved the submitted version.
