# Supplementary material for: Prognostic value of post-discharge depression in patients recently hospitalized with acute heart failure
Source: Front Cardiovasc Med. 2022 Aug 2;9:858751. doi: 10.3389/fcvm.2022.858751 (PMC9378836; doi:10.3389/fcvm.2022.858751)
Supplement: Supplementary file 1 [file Data_Sheet_1.docx]

Supplementary Material

1. S-Figure 1. The depression and all-cause mortality rates in patients with different body mass indexes and age.
2. S-Table 1. Multiple imputations were used for the following variables in the adjusted model.
3. S-Table 2. Multiple logistic regression analysis of predictive factors associated with post-discharge depression (conducted with complete case analysis).
4. S -Table 3 The associations of depression with all-cause mortality and the composite event (conducted with complete case analysis).
5. S-Table 4. The associations of baseline PHQ-9 scores (per scale point increase) with all-cause mortality and the composite event.
6. Details of the log transformation and standard normal transformation for natriuretic peptide (NPs).
7. The authors would like to acknowledge the 73 HERO participating hospitals in the HERO study.

# S-Figure 1. The depression and all-cause mortality rates in patients with different body mass indexes and age.

# S-Table 1. Multiple imputations were used for the following variables in the adjusted model.

Multiple imputations (Markov chain Monte Carlo method) were used conducted to handle the missing data, and a total of 40 imputed datasets were generated. Maximum iterations: 10.

| Variables | Missing rate, n (%) |
| --- | --- |
| Serum creatinine(eGFR) | 267 (7.7) |
| Body mass index | 259 (7.5) |
| Serum sodium | 162 (4.7) |
| Hemoglobin | 132 (3.8) |
| BNP/NT-proBNP | 686(19.8) |
| In-hospital LVEF | 1432(41.4) |
| Systolic blood pressure | 10(0.3) |
| Current smoker | 19 (0.5) |
| diabetes | 13 (0.4) |
| Coronary heart disease | 28(0.8) |
| ACEI/ARB/ARNI use at discharge | 36 (1.0) |
| Beta-blockers use at discharge | 29 (0.8) |
| MRA use at discharge | 28 (0.8) |
| Statin use at discharge | 34 (1.0) |

S-Table 2. Multiple logistic regression analysis of predictive factors associated with post-discharge depression (conducted with complete case analysis).

| Model Covariates | Odds ratio(95%CI) | p value |
| --- | --- | --- |
| Age(y) | 1.003 (0.989-1.018) | 0.680 |
| Sex (female vs male) | 1.547 (1.114-2.148) | 0.009 |
| BMI, Kg/m2 | 0.978 (0.936-1.021) | 0.313 |
| Diabetes | 1.587 (1.093-2.304) | 0.015 |
| COPD | 1.600 (0.962-2.661) | 0.070 |
| eGFR<60 mL/min/1.73m^2^ | 1.018 (0.716-1.447) | 0.922 |
| Anemia* | 1.096 (0.791-1.520) | 0.581 |
| Hyponatremia# | 1.302 (0.864-1.962) | 0.208 |
| NYHA (IV versus Ⅲ) | 1.261 (0.909-1.750) | 0.164 |
| Lg NT-proBNP | 1.262 (0.978-1.628) | 0.073 |
| ACEI/ARB/ARNI use at discharge | 0.749 (0.540-1.039) | 0.164 |
| Beta-blocker use at discharge | 0.901 (0.649-1.252) | 0.535 |
| Statin use at discharge | 1.012 (0.716-1.430) | 0.946 |
| Hospital level (tertiary vs secondary) | 0.749 (0.508-1.100) | 0.105 |

*Anemia, hemoglobin <13g/dL in male or hemoglobin <12g/dL in female. # Hyponatremia, serum sodium concentration <135mmol/L.

# S -Table 4. The associations of depression with all-cause mortality and the composite event (conducted with complete case analysis).

| Events | Adjusted HR (95%CI) | p value |
| --- | --- | --- |
| All-cause mortality | 2.41(1.91-3.04) | <0.001 |
| Composite event (death or HF rehospitalization) | 1.87 (1.61-2.19) | <0.001 |

Adjusted variables: age, sex, body mass index, systolic blood pressure, current smoker, diabetes, chronic obstructive pulmonary disease, coronary heart disease, anemia, estimated glomerular filtration rate, serum sodium concentration, in-hospital left ventricular ejection fraction (<40%, 40%-49%, ≥50%, and unavailable), New York Heart Association class, use of renin-angiotensin system inhibitors at discharge, use of β-blockers at discharge, use of mineralocorticoid receptor antagonists at discharge, use of statin at discharge, hospital levels. Anemia was defined as hemoglobin <13g/dL in male or hemoglobin <12g/dL in female. HR, hazard ratio; CI: confidence interval.

# S-Table 5. The associations of baseline PHQ-9 scores (per scale point increase) with all-cause mortality and the composite event

| Events | Adjusted HR (95%CI) | p value |
| --- | --- | --- |
| All-cause mortality | 1.076(1.061- 1.091) | <0.001 |
| Composite event (death or HF rehospitalization) | 1.046(1.036-1.056) | <0.001 |

Adjusted variables: age, sex, body mass index, systolic blood pressure, current smoker, diabetes, chronic obstructive pulmonary disease, coronary heart disease, anemia, estimated glomerular filtration rate, serum sodium concentration, in-hospital left ventricular ejection fraction groups(<40%, 40%-49%, ≥50%), New York Heart Association class, use of renin-angiotensin system inhibitors at discharge, use of β-blockers at discharge, use of mineralocorticoid receptor antagonists at discharge, use of statin at discharge, hospital levels. Anemia was defined as hemoglobin <13g/dL in male or hemoglobin <12g/dL in female. HR, hazard ratio; CI: confidence interval.

# Details of the log transformation and standard normal transformation for natriuretic peptide (NPs).

- NT-proBNP levels were measured in 1890 patients during hospitalization. The distribution of NT-proBNP values was skewed (median, 2817.76; IQR, 869.33, 7074.25).
- After log transformation (the base was 10), the values of LgNT-proBNP were normally distributed (mean±SD, 3.3291±0.69805).
- According to the formula for standard normal transformation [z score=(x-mean)/SD], where standard LgNT-proBNP=(LgNTproBNP- 3.3291)/0.69805. The mean(±SD) of standard LgNT-proBNP was 0.00(±1.00).
- Of 1566 patients without NT-proBNP, BNP levels were measured in 880 patients during the hospitalization. The processes of log transformation and standard normal transformation were similar to NT-proBNP. The mean(±SD) of standard LgBNP values in the 880 patients was also 0.00(±1.00).
- Subsequently, we combined the values of standard LgNT-proBNP and standard LgBNP as a new variable, NPs. The table below shows the median and IQR during log and standard normal transformations.

# The authors would like to acknowledge the 73 HERO participating hospitals in the HERO study.

| Central Hospital of the Yellow River: Liwei Jin. | Changyuan Hospital of Traditional Chinese Medicine: Fei Wang, Luping Dong. |
| --- | --- |
| County People’s Hospital of Anyang: Jinfu Yang, Zhixiang Han. | County People’s Hospital of Puyang: Shanyong Ding, Xiaohua Yang. |
| First Affiliated Hospital of Nanyang Medical College: Zhanhai Zhang, Sheng Yang. | First Affiliated Hospital of Xinxiang Medical College: Guoan Zhao, Hui Liu. |
| Hebi Hospital of Traditional Chinese Medicine: Shuxia Zhang. | Henan provincial Chest Hospital: Kejun Huang, Li Li. |
| Huaihe Hospital of Henan University: Guanchang Cheng, Qilin Wan. | Jiyuan People’s Hospital: Zhiyong Zhao, Huixuan Liu. |
| Kaifeng Central Hospital: Lei Qin, Jieyun Liu. | Luohe Second People's Hospital: Huixin Li, Guang Yang. |
| Luohe Central Hospital: Dongliang Liu, Haoran Wang. | Luoyang Central Hospital Affiliated to Zhengzhou University: Shouyan Zhang, Hao Wang. |
| Nanyang Central Hospital: Shouzhong Yang, Wenyu Shen. | Pingdingshan First People's Hospital: Xinjie Duan, Tiexu Zhang, Yaohui Wang. |
| Puyang Oilfield General Hospital: Hengliang Wang, Yuanyuan Nie. | Sanmenxia Central Hospital: Huiyu Wang, Yong Zhang. |
| Shangqiu First People's Hospital: Xuesheng Xu. | The First Affiliated Hospital of Henan University of TraditionalChinese Medicine: Huaimin Guan, He Wang. |
| The First Affiliated Hospital of Henan University of Science and Technology: Xuming Yang, Laijing Du. | The First Affiliated Hospital of Zhengzhou University: Jianzeng Dong, Ling Li, Li Li. |
| The First People's Hospital of Gushi: Zelin Zhang, Ling Liang. | The First People's Hospital of Lingbao: Wanke Li, Jinwang Song. |
| The First People's Hospital of Ruzhou: Junxing Hu, Zhanpo Ren. | The First People's Hospital of Xinmi: Xiaolei Li, Jie Dou. |
| The People’s Hospital of Anyang City: Wenjian Jia, Hongxia Sun. | The People’s Hospital of Changge: Fujian Lu, Baojian Shi. |
| The People’s Hospital of Changyuan: Guorui Hou, Guangyan He. | The People's Hospital of Dancheng: Tianming Yu, Fengbo Li. |
| The People's Hospital of Dengfeng: Hongxu Geng, Jianli Yan. | The People's Hospital of Dengzhou: ChuanpingWang, Ying xiao. |
| The People's Hospital of Fangcheng: Yintao Qiao, Dawei Liu. | The People's Hospital of Gongyi: Tianmin Du, Junjie Fu. |
| The People's Hospital of Guangshan: Qi Wang, Xin Zheng. | The People’s Hospital of Huaiyang: Changheng Han, Tingchun Chen. |
| The People's Hospital of Huaxian: Wenli Liu, Jingjing Wang. | The People's Hospital of Jiaozuo City: Haijun Zheng. |
| The People's Hospital of Linzhou: Zhoushun Qin, Zhili Wang. | The People's Hospital of Lushan: Dalao Ding, Zhigang Li. |
| The People's Hospital of Lushi: Zhanjiang Wang. | The People's Hospital of Luyi: Yuanxun Xu. |
| The People's Hospital of Nanzhao: Yuchun Li. | The People's Hospital of Pingyu: Xiang Xue, Heng Luo. |
| The People’s Hospital of Qixian: Shengke Zhu. | The People’s Hospital of Qinyang: Xiaowen Ma, Yanli Liang. |
| The People's Hospital of Shangcai: Dechen Wang. | The People’s Hospital of Shangcheng: Chenhui Xiong, Zhenhua Li. |
| The People's Hospital of Shangshui: Zijun Zhu, Donghan Yao | The People's Hospital of Taikang: Lizhi Lu, Xiaoqian Wu. |
| The People's Hospital of Tongxu: Dayin Yu. | The People's Hospital of Weishi: Fang Zou, Hongen Liu. |
| The People's Hospital of Wenxian: Xiaoli Ji, Fuqing Zhang. | The People's Hospital of Xiangcheng: Qingfan Zhang, Lina Hu. |
| The People's Hospital of Xichuan: Hua Wang, Xijuan Ren. | The People’s Hospital of Xihua: Chuntong Wang, Yongwei Wang. |
| The People’s Hospital of Xincai: Yuhua Song. | The People’s Hospital of Yexian: Jie Yang. |
| The People's Hospital of Yongcheng: Changming Tian, JingWang, Wei Li. | The People's Hospital of Yuzhou: Yuting Geng, Qiongyao Pan. |
| The People's Hospital of Zhecheng: Zhenfu Zhao. | The People's Hospital of Zhenping: Xiao Liu, Lexing Wang. |
| The People's Hospital of Zhongmou: Xiuzhen Kong, Xia Li. | The Second Affiliated Hospital of Zhengzhou University: Lihua Zhang, Qing Zhi. |
| Xinxiang Central Hospital: Zhifang Wang, Shuhong Su. | Xinyang Central Hospital: Junqiang Yuan, Zhihai Li. |
| Xuchang Central Hospital: Shaoyu Chen, Mingshuan Guo. | Yuzhou Central Hospital: Qinfeng Su, Jing Zhao. |
| Zhengzhou Hospital of Traditional Chinese Medicine: Qingju Luo, Chenjun Lu. | Zhengzhou NO.7 People's Hospital: Yiqiang Yuan, Qiong Huang. |
| Zhoukou Central Hospital: Juntang Zhang, Qian Zhou. | Zhoukou People's Hospital: Chunzhi Xu. |
| Zhumadian Central Hospital: Lingxia Guan, Jicheng Liu. |  |
